# Electromagnetic–Acoustic Sensing for Biomedical Applications

**DOI:** 10.3390/s18103203

**Published:** 2018-09-21

**Authors:** Siyu Liu, Ruochong Zhang, Zesheng Zheng, Yuanjin Zheng

**Affiliations:** School of Electrical and Electronic Engineering, Nanyang Technological University, Singapore 639798, Singapore; sliu023@e.ntu.edu.sg (S.L.); rzhang009@e.ntu.edu.sg (R.Z.); ZESHENG001@e.ntu.edu.sg (Z.Z.)

**Keywords:** electromagnetic-acoustics, photoacoustic, thermoacoustic, biomedical sensing

## Abstract

This paper reviews the theories and applications of electromagnetic–acoustic (EMA) techniques (covering light-induced photoacoustic, microwave-induced thermoacoustic, magnetic-modulated thermoacoustic, and X-ray-induced thermoacoustic) belonging to the more general area of electromagnetic (EM) hybrid techniques. The theories cover excitation of high-power EM field (laser, microwave, magnetic field, and X-ray) and subsequent acoustic wave generation. The applications of EMA methods include structural imaging, blood flowmetry, thermometry, dosimetry for radiation therapy, hemoglobin oxygen saturation (SO_2_) sensing, fingerprint imaging and sensing, glucose sensing, pH sensing, etc. Several other EM-related acoustic methods, including magnetoacoustic, magnetomotive ultrasound, and magnetomotive photoacoustic are also described. It is believed that EMA has great potential in both pre-clinical research and medical practice.

## 1. Introduction

Electromagnetic (EM) wave-based imaging and sensing methods have been widely used in clinical practice. These techniques play a vital role in physiological sensing, clinical diagnosis, image-guided treatment, and patient management. Although tremendous progress has been achieved, there are still some fundamental limitations to these EM techniques. Among them, X-ray computed tomography (CT) is considered to be the clinical gold standard technique for medical diagnosis [1]. Although X-rays provide deep penetration depth and great imaging resolution, the associated ionizing radiation damage limits its application. Optical imaging, including optical coherence tomography (OCT) [2], diffuse optical tomography [3], near-infrared spectroscopy (NIRS) [4], and fluorescence imaging [5], could provide high spatial resolution and high specificity for surface investigation. Nevertheless, accurate optical focusing relies on the ballistic propagation of photons within biological tissue, limiting the imaging depth to a few hundred micrometers (mean free path of photons) [6]. Microwave-based sensing techniques normally probe the dielectric properties of the biological tissues in sub-GHz to several GHz spectrum range, which provides high-contrast information on abnormal tissues [7]. On the other hand, its longer wavelength limits its spatial resolution to several mm, even processed by advanced beamforming techniques [8]. Beyond the EM imaging modalities introduced above, there are some other types of sensing techniques, such as magnetic resonance imaging (MRI) [9], magnetic induction tomography (MIT) [10], electrical impedance tomography (EIT) [11], hall effect imaging (HEI) [12], magnetic resonance electrical impedance tomography (MREIT) [13], etc. that obtain dielectric properties by using an alternating magnetic field or varying current injections. All of these EM-based modalities show their internal limitations and cannot meet the stringent requirements (high sensitivity, deep penetration, high spatial resolution, real time, low cost, etc.) of modern biomedical applications.

Electromagnetic–acoustic (EMA) techniques have emerged with the goal of overcoming the above limitations. By combining the merits of both EM wave-based (e.g., high-contrast/specificity) and acoustic wave-based (e.g., high resolution and deep penetration) sensing modalities, EMA techniques are considered to provide superior imaging performance than traditional EM methods. In EMA, the biological tissue or exogenous contrast agent is irradiated and heated by a high-power EM field. The localized temperature elevation causes the transient thermoelastic expansion of the tissue, and finally ultrasonic wave emission. The image contrast is determined primarily by tissue EM properties, while image resolution is primarily provided by the ultrasound wave. Based on the types of the EM excitation employed (laser, microwave, magnetic field, X-ray, respectively), EMA can be classified into light-induced photoacoustics (PA), microwave-induced thermoacoustics (TA), magnetic-modulated thermoacoustics (MMTA), and X-ray-induced thermoacoustics (XTA), correspondingly.

As the mainstream EMA technique, the PA imaging and sensing technique is based on the photoacoustic effect, which was first discovered by Alexander Graham Bell in 1880 [14]. Although the study of PA technique started early in the 1970s, PA only began to be investigated for biomedical applications in the mid-1990s [15,16]. At the same time, a microwave-based TA technique was proposed by Kruger’s and Wang’s groups [17,18,19,20,21]. Inspired by the PA and TA techniques above, it is expected that acoustic waves should also be induced by other EM excitation, e.g., X-ray and radio-frequency magnetic excitation, leading to the emergence of XTA [22,23] and MMTA [24,25]. With the publication of the above reports, more and more researchers became aware of the advantages of EMA and directed their efforts to improving EMA techniques. EMA techniques witnessed a tremendous growth in terms of development of system [26,27,28,29,30,31,32], reconstruction algorithm [33,34,35,36,37], functional imaging capabilities [38,39,40,41,42,43,44,45,46], and in vivo applications [47,48,49,50,51,52,53]. Therefore, a short review paper like this can only cover a limited amount of the existing literature. This article is intended to provide an overview of the EMA techniques (covering PA, TA, MMTA, and XTA) and focus on the fundamental principles of each technique (electromagnetically and acoustically), along with the major biomedical applications in the last couple of years.

## 2. Principles

### 2.1. EM Absorption and Heating

The fundamental principles of the EMA can be simply described: Biological tissue or exogenous contrast agent absorbs EM radiation energy partially, leading to localized heating, temperature elevation, and thermoelastic expansion, which launches ultrasonic wave propagation outwards.

Interactions between EM radiation and biological tissue have been widely studied, such as Rayleigh scattering, Raman scattering, absorption, etc. [54,55,56]. The most important interactions with regard to EMA techniques are the EM absorption and scattering properties. EM absorption determines the origin of acoustic generation and also the sensitivity, while the scattering degrades the sensing resolution, especially for PA. Although all EMA techniques share the same acoustic propagation mechanism with the traditional ultrasound techniques, the principles of EM absorption and heating generation are slightly different.

#### 2.1.1. Light-Induced Photoacoustics (PA)

In biological tissues, light transfer is dominated by scattering and diffusion [57]. Beyond the transport mean free path, photons enter into the diffusive regime and undergo significant scattering, making focusing based optical sensing techniques become ineffective. Therefore, pure optical techniques with diffused light can only achieve a resolution of about 1 cm [58]. On the other hand, in the medium containing strong photo absorbers (e.g., hemoglobin and pigment), although light scattering broadens and attenuates light greatly, the generated PA wave provides better resolution than the optical wave, because ultrasonic scattering is two to three orders of magnitude weaker than optical scattering [59].

**Figure 1:**
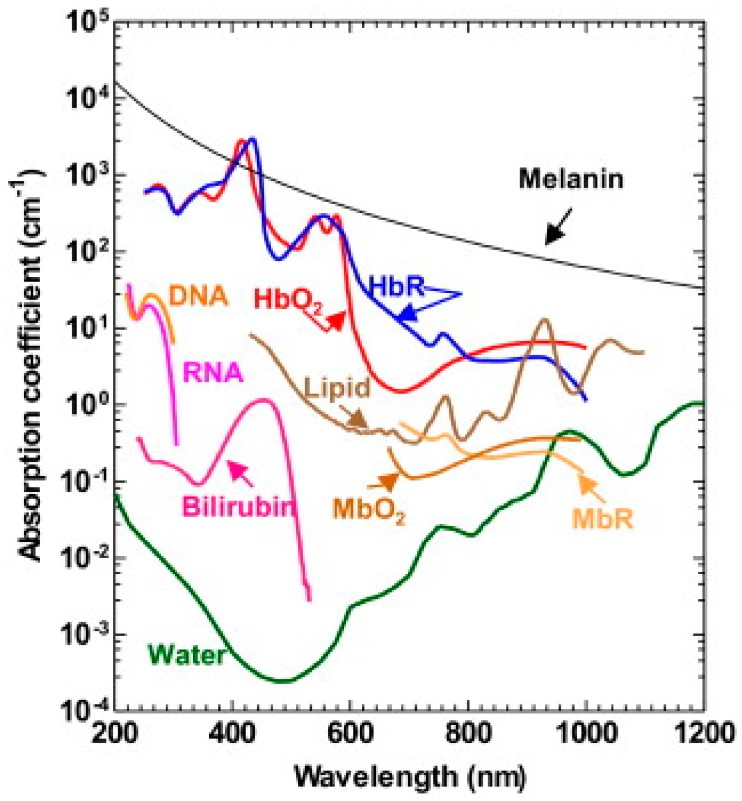
Absorption spectrum of major contrast agents in biological tissue from 200 to 1200 nm in wavelength, including melanin, oxyhemoglobin (HbO_2_), deoxyhemoglobin (HbR), DNA and RNA, lipid, bilirubin, oxy-myoglobin (MbO_2_), reduced myoglobin (MbR), and water. Reprinted with permission from [68].

The most commonly photo absorbers for PA imaging and sensing can be classified into two categories based on their primary absorbing wavelengths: (1) in the visible regions (400–700 nm), the primary absorbers for PA include hemoglobin [40,60], cytochrome [61], melanin [62], DNA/RNA [49,63], myoglobin [64]; (2) in the near-infrared region (700–1400 nm), lipid [65,66], glucose [45,67] are also strong absorbers for PA emission. Figure 1 summarizes the absorption spectrum of common endogenous contrasts [68]. Among these, hemoglobin is most commonly used for PA imaging and sensing, where the absorption contrast between blood vessels and background tissues is more than 100. In addition, the difference of absorption spectrum between oxygenated and deoxygenated hemoglobin makes PA intrinsically suited for spectroscopic detection, leading to wide applications of PA functional imaging.

Two types of laser sources can be employed to generate PA signals: short pulses and intensity-modulated continuous-wave (CW) EM laser. Although the CW-based PA method shows wide advantages including compact system size, low cost, low power consumption, portability, short laser pulse (nanosecond, especially) is still preferred to provide a higher signal-to-noise ratio (SNR) and direct detection of the distance of the source through the time-resolved PA signals. Following a short laser pulse excitation with light fluence of ϕ(r,t), the heating function H(r,t), defined as absorbed energy per unit time and per unit volume, is expressed as:

(1) $$H(r,t)=\mu_{a}(r)\phi (r,t),$$

which serves as the source term to induce PA generation. In the equation, the absorption coefficient $\mu_{a}(r)$ is the probability of photon absorption per unit path length.

#### 2.1.2. Microwave-Induced Thermoacoustics (TA)

When the laser excitation is replaced by microwave, the technique is called TA. Since wavelengths of microwaves (0.3–3 GHz) are comparable to the size of normal biological tissue, such as the breast, the resolution of the microwave-based pure EM technique is greatly limited. However, the generated TA wave with sub-millimeter wavelength can enable a higher resolution, with negligible acoustic scattering and attenuation.

The TA properties of biological tissues are related to the physiological nature of their dielectric properties, especially the relative conductivity. In this sense, when microwave energy delivered in a pulse that is short enough (thermal confinement), the resulting heating function is:

(2) $$H(r,t)=\sigma (r)\langle E^{2}(r,t)\rangle ,$$

in which σ(r) and E(r,t) are the conductivity distribution and electrical field strength, respectively. 〈…〉 represents short time average operation. At 3 GHz, the electrical conductivity for normal muscle is more than 7 times higher than that of the fat tissue [69], while most of the other soft tissues have the conductivity in between those for muscle and fat. This wide range of values among various biological tissues can provide a high contrast for TA sensing. Furthermore, the tissue dielectric properties were found to highly correlate to their water content, enabling a higher TA generation from cancerous tumor (e.g., breast tumor tissue) than surrounding tissue. Therefore, TA may potentially be used to detect early-stage cancers.

#### 2.1.3. X-ray-Induced Thermoacoustics (XTA)

Analogously to PA and TA, XTA is an important EMA sensing technique for medical applications, especially associated with CT imaging and radiotherapy. Since X-rays carry enough energy to ionize atoms and disrupt molecular bonds, the X-ray radiation is ionizing and harmful to biological tissue [1]. A high radiation dose over a short period of time can induce radiation sickness, and also give an increased risk of cancer. Therefore, the radiation dose exposed to human body during the process of diagnosis/therapy should be controlled carefully. With the aim of dose monitoring, XTA is developed as a powerful sensing tool.

During X-ray radiation, X-rays can be absorbed by inner-shell electrons and generate photoelectrons [70,71]. The Auger electrons and generated photoelectrons transfer part of their kinetic energy to the surrounding medium to reach a thermal equilibrium. Such electron–phonon interaction-induced energy transfer leads to temperature increase and heat deposition in the biological tissue, as follows:

(3) $$H(r,t)=\mu_{x}(r)F(r,t),$$

where $\mu_{x}(r)$ is the absorption coefficient distribution of X-ray for biological tissue, and F(r,t) represents the X-ray fluence at location r and time t. To induce efficient XTA generation, a short X-ray pulse is employed (e.g., 5 μs in [23]). Such an XTA wave can be detected by an ultrasound transducer for dose calculation and mapping, which will be discussed more in the Section 3.4.

#### 2.1.4. Magnetic Modulated Thermoacoustics (MMTA)

In TA, the penetration depth for microwave is limited to be within several centimeters [72], which is determined by the significant EM absorption of high-water-content tissue. By employing radio frequency (RF) magnetic stimulation, MMTA is a more energy-efficient solution for potentially deeper penetration. Since the human body is non-magnetic and responds to magnetic field nearly as free space, the magnetic field can penetrate tissues deeper without any absorption attenuation [24].

Heating of magnetic nanoparticles or its dispersed ferrofluid with magnetic field is thought to be caused by a combination of the hysteresis effect, Brownian relaxation, and Néel relaxation [73]. The area within the hysteresis loop illustrates the magnetic energy delivered in the form of heat to nanoparticles during the reversal of magnetization. By stimulating the alternating magnetic field (AMF), the heat function can be calculated as follows: [25]

(4) $$H(r,t)=\frac{1}{2}\mu_{0}\chi (r)\langle H^{2}(r,t)\rangle \frac{\omega_{0}{}^{2}\tau }{1+{\left(\omega_{0}\tau \right)}^{2}},$$

where $\mu_{0}$ is the permeability of free space, $\omega_{0}$ is the carrier frequency of magnetic field, τ is the effective relaxation time of the nanoparticles, and χ(r) is the localized magnetic susceptibility, which maps the nanoparticle distribution inside biological tissue. Therefore, the magnetic-based EMA approach is immune from large permittivity of human tissues for depositing electric energy inside tissues, enabling a high-SNR tracking of nanoparticle motion. Such a feature can be employed to monitor nanoparticle accumulation and magnetic hyperthermia [74].

### 2.2. EM Acoustic Generation

To generate EMA signals efficiently, two conditions, referred to as thermal and stress confinements, must be satisfied [75]. Firstly, the EM pulse used to stimulate the biological tissue should have a pulse width τ smaller than the characteristic thermal relaxation time $\tau_{\mathrm{th}}$ so that thermal diffusion can be neglected. Such a condition is called the thermal confinement condition. Normally, the time scale for the heat dissipation in biological tissue by thermal conduction is about several milliseconds. In EMA, to generate efficient MHz ultrasound signals, the time duration of the EM pulse is always from several nanoseconds to microseconds, which is much less than the required thermal confinement relaxation time. On the other hand, the EM pulse width should also be smaller than the time for the stress to exit the heated region, which is commonly known as stress confinement [59]. When this condition is satisfied, high thermoelastic pressure in the tissue can build up rapidly. For example, to achieve a spatial resolution of 100 μm, the acoustic relaxation time is more than several tens of millisecond. Therefore, the acoustic confinement condition can also be easily satisfied.

Upon the fulfillment of the thermal confinement and stress confinement, thermal diffusion and fractional volume expansion can be negligible. The local pressure rise $p_{0}(r)$ immediately after EM absorption can be derived as follows: [75]

(5) $$p_{0}(r)=-\frac{\beta }{c}\frac{\partial }{\partial t}H(r,t),$$

in which β is the thermal coefficient of volume expansion, and C is the specific heat capacity of the tissue medium. Therefore, the initial EMA pressure is directly related to the spatial distribution of EM absorption. Such acoustic transient pressure inside the tissue acts as the initial condition in the general acoustic wave equation:

(6) $$\nabla^{2}p(r,t)-\frac{1}{c^{2}}\frac{\partial^{2}}{\partial t^{2}}p(r,t)=-\frac{\beta }{C}\frac{\partial }{\partial t}H(r,t).$$

The generated EMA wave propagates through the sample and is detected by an ultrasonic transducer or transducer array at the tissue boundary. Solved by three-dimensional Green function method [76], the EMA signal picked up by an ideal point ultrasound transducer at $r_{d}$ can be written as [57]

(7) $$p(r_{d},t)=\frac{1}{4\pi c^{2}}\frac{\partial }{\partial t}\int dr\frac{1}{\left|r_{d}-r\right|}H(r,t-\frac{\left|r_{d}-r\right|}{c}).$$

Equation (7) tells us that the detected acoustic pressure at spatial location $r_{d}$ and time t comes from an acoustic source integrated over a spherical shell centered at $r_{d}$ with a radius of ct. Such a direct relationship between generated acoustic pressure and the capabilities of EM absorption permits the structural imaging and quantification of various physiological parameters such as the oxygenation of hemoglobin. In addition, in the process of EM heating, the transient temperature elevation is about several millidegrees Celsius [26], and thus will not change the properties of the biological tissue under study, making EMA techniques suitable for wide biomedical sensing applications.

Up to now, we have discussed the generation and propagation of EMA pressure waves in tissue with different EM excitation. In the following sections, we will review the applications of EMA techniques in biomedicine, including imaging, sensing, and therapeutic-related methods.

## 3. Biomedical Sensing Application

In this section, several biomedical applications of EMA techniques will be reviewed. These applications are majorly focused on the biomedicine field, including structural imaging (brain imaging, whole-body imaging, and molecular imaging), blood flowmetry, thermometry, dosimetry for radiation therapy, hemoglobin oxygen saturation (SO_2_) sensing, fingerprint imaging and sensing, glucose sensing, and pH sensing.

### 3.1. Structural and Molecular Imaging

#### 3.1.1. Brain Imaging

**Figure 2:**
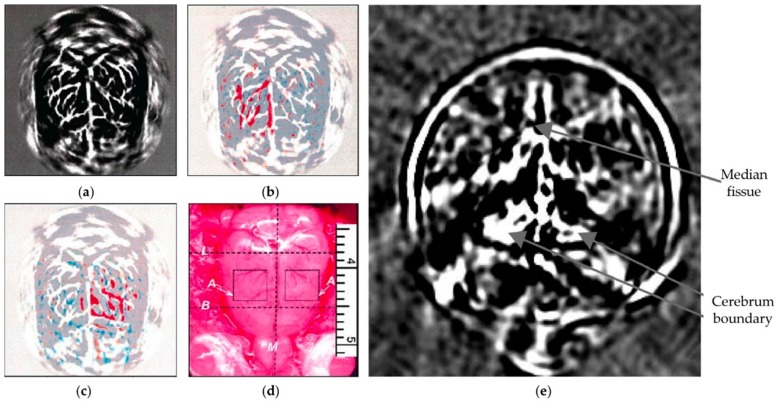
(**a**) PA brain imaging of nude mouse; (**b**,**c**) PA images corresponding to left-side and right-side whisker stimulation, respectively; (**d**) open-skull photograph of the rat cortical; (**e**) TA imaging of rhesus monkey brain. Reprinted with permission from [26,80].

Brain imaging has become one of the most important breakthroughs in cognitive neuroscience; it will not just unlock the mystery of how brain works, but also provides guidance for better diagnostics and therapeutics of neural diseases. By using different implementations and focusing mechanism, PA brain imaging methods from microscopic to macroscopic scales have been extensively reported [26,32,77,78,79,80]. Figure 2a shows the imaging results obtained by photoacoustic computed tomography (PACT), a major tool for PA imaging, in which the ultrasound transducer is driven by a computer-controlled motor to circularly scan around the rat head in the imaging plane. By stimulating the left-side and right-side whisker, corresponding differential PA images are illustrated in Figure 2b,c, which agree well with the vascular pattern shown in Figure 2a. Such elevation of PA amplitude in the stimulated regions indicates increased cortical neural activity in response to whisker stimulation. Later, instead of imaging cortical surface, PA imaging of the whole mouse brain has been demonstrated based on the structural contrast from other chromophores [78].

Providing different contrast information, TA brain imaging has also been demonstrated through rhesus monkey skulls [80]. Benefiting from the low absorption and diffraction from the skull, the TA method is considered to provide superior penetration compared to the laser-based PA imaging method. Figure 2e shows a tomographic image of the rhesus monkey brain made by the TA method. The gray matter and cerebrospinal fluid (CSF) appear darker due to large EM absorption (more water), whereas the white matter shows less TA generation (less water). In addition, the median fissure and the boundary between the cerebra and cerebella can also be explicitly imaged.

A long-standing challenge of brain imaging is eliminating the ultrasonic distortion provided by the skull. Such phase distortion can be alleviated by the low-pass-filtering [80]; however, this sacrifices the imaging resolution. A potential solution is to use geometric measurements of the skull from CT or MRI to quantify the aberration effect and then compensate for the phase shift. A more detailed review of PA/TA brain imaging can be found in [81].

#### 3.1.2. Whole-Body Imaging

Besides brain imaging at the organ level, EMA techniques are also suitable for whole-body imaging for small animals. Small-animal models are widely used in biomedical research and the whole-body imaging of small animals is a critical step in pre-clinical studies.

**Figure 3:**
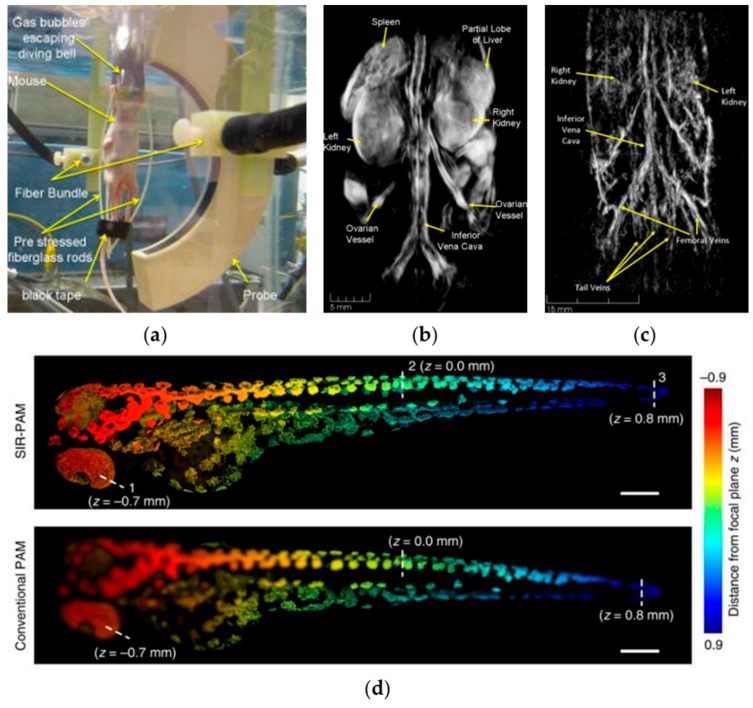
(**a**) Photograph of the whole-body PA imaging of a nude mouse; (**b**) whole-body PA imaging of a nude mouse with 755 nm excitation; (**c**) whole-body PA imaging of a nude mouse with 1064 nm excitation; (**d**) whole-body PA images of zebrafish embryos encoded with imaging depth. Reprinted with permission from [82,83].

Brecht et al. designed a small-animal PACT system in which optical illumination is provided by two fiber bundles from opposite directions, while PA signals are received by a 64-element curved array [82] (see the diagram of the system in Figure 3a). Figure 3b shows a PA image of a nude mouse acquired at 755 nm excitation wavelength. The left and right kidneys, spleen, and a partial lobe of the liver can also be easily visualized. At another exciting wavelength, 1064 nm, with strong absorption in both arterial and venous vasculature, the descending aorta, the abdominal aorta, and its bifurcation into the femoral veins can be clearly imaged.

In [83], whole-body images of living zebrafish embryos were obtained with motionless volumetric PA microscopy. Figure 3d depicts the whole zebrafish larva, with imaging depths encoded in color. With spatially invariant resolution, structures throughout the whole fish can be clearly resolved, whereas the imaging in conventional PA microscopy is blurry [83].

Some other techniques for developing whole-body PA imaging include spectroscopic whole-body PA imaging [84], long-term distribution of nanoparticle and drug delivery [85], and whole-body imaging of adult zebrafish [86]. A more detailed review of whole-body imaging of small animals can be found in [87].

#### 3.1.3. Molecular Imaging

In the EMA imaging method, endogenous PA contrasts (e.g., DNA/RNA in cell nuclei and hemoglobin in blood cells) and TA contrasts (e.g., high water content tissues) are normally employed as the EMA source and contrast provider. However, they may lack the requisite biological specificity for diagnosing applications.

EM absorption-based EMA techniques (including PA, TA, and MMTA) can be easily extended to the molecular imaging of pathologically changed tissues (e.g., cancer cells), with the implementation of EM-absorbent biomarkers, in which a wide range of nanoparticles and dyes are suitable for PA molecular imaging and magnetic nanoparticles are suitable for TA and MMTA imaging [88,89,90,91,92]. If the exogenous contrast agents are properly conjugated to bioactive peptides, antibodies, proteins, and hormones, imaging contrast would be greatly improved by up to 30 dB [93]. In addition, the molecular imaging also enables the visualization of the cellular function and tracks the dynamic activity of the molecular process.

In particular, MMTA is intrinsically a molecular imaging method. By employing magnetic nanoparticles, an EMA wave can only be generated from the nanoparticles, whereas the background biological tissue acts as the free space without any magnetic response [24]. In addition, such magnetic nanoparticles can be easily modulated by the external magnetic field to realize some attractive capabilities, e.g., magnetic saturation [25]. Several review papers about nanoparticle-based PA/TA molecular imaging can be found in [94,95].

### 3.2. Flowmetry

**Figure 4:**
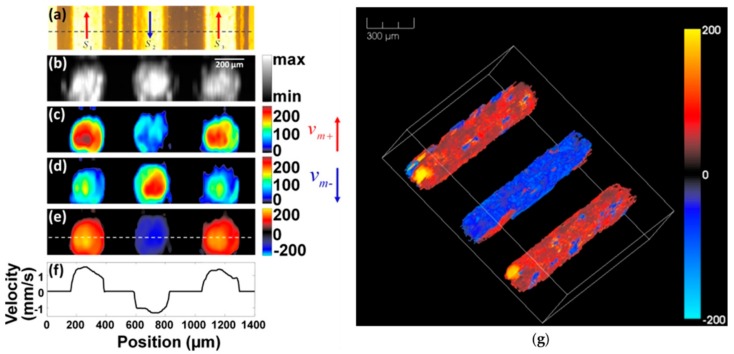
PA Doppler flowmetry. (**a**) PA Doppler bandwidth as a function of flow velocity; (**b**–**f**) PA flow imaging at different velocities and directions; (**g**) a 3D visualization of the PA flow imaging. Reprinted with permission from [101].

Blood flowmetry techniques can detect abnormal flow within an artery or blood vessel. This can help to diagnose and treat a variety of conditions, including blood clots and poor circulation [96]. As opposed to back-scattered waves used in pure ultrasonic and optical method, EMA techniques rely on EM absorption, offering a high contrast between vasculature and the surrounding tissues. A variety of detection mechanisms have been reported, including Doppler Effect [97], density tracking [98], transit time of particles [99], and flash/replenishment-based photoconversion [100]. Figure 4 shows the results of transverse-flowing imaging based on the PA Doppler bandwidth broadening, in which the PA spectrum is demonstrated to be broadened by the blood flow [101]. Based on the measured Doppler bandwidth from Figure 4a,b, the transient blood velocity can be quantified and visualized (as shown in Figure 4c,g), in which positive flow direction (the same direction as the acoustic wave propagation) is shown in red, and the negative flow is shown in blue. In Figure 4f, it can be clearly seen that the measured flow speed at the boundary is relatively low, which is consistent with the parabolic velocity model in blood vessels.

Up to now, PA flow imaging has not yet been applied clinically or pre-clinically. One of the major challenges is the limited penetration depth of light in opaque biological tissues, while conventional ultrasound color Doppler imaging could be used in deep tissues. The combination of high-sensitivity nanoparticles and TA/MMTA may drive the clinical translation of EMA techniques with high accuracy and stability.

### 3.3. Thermometry

EMA techniques including PA/TA/MMTA have been demonstrated to noninvasively measure tissue temperature with high sensitivity, deep penetration, and real-time capability [24,39]. Such deep-temperature sensing techniques have applications in thermotherapy (e.g., HIFU ablation, radiofrequency ablation, photothermal therapy, etc.), in which heat is used to ablate abnormal tissue such as tumors. During the process of thermotherapy, to ensure only the cancerous cells are destroyed while leaving surrounding healthy tissue unaffected, such thermometry or dosimetry with deep penetration is of particular importance [102,103].

**Figure 5:**
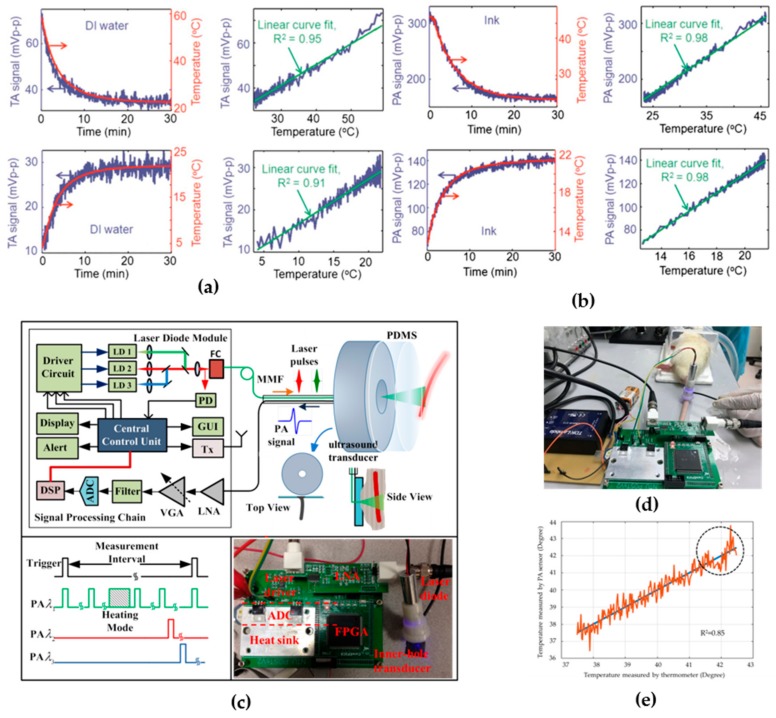
(**a**) TA temperature sensing of deionized water; (**b**) PA temperature sensing of ink; (**c**) portable PA device for temperature measurement; (**d**) in vivo testing of the portable PA device; (**e**) results of PA temperature measurement in vivo. Reprinted with permission from [39,106].

The principles for temperature sensing-based EMA techniques are quite straightforward. The volume expansion coefficient β and the speed of sound c in Equation (5) are both temperature-dependent and linearly proportional to the environmental temperature between 10 and 55 °C [104]. Therefore, the Grueneisen parameter, and thus initial acoustic pressure $p_{0}$ can be directly used for in vivo temperature sensing and mapping. Figure 5a,b shows the TA and PA amplitude dependence of temperature elevation, respectively. The sensing accuracy is estimated to be about 0.15 °C and the temporal resolution is as short as 2 s [39]. With spatial resolution provided by mechanical scanning or ultrasound linear array [104,105], temperature thermal mapping is also available, which provides not only temperature elevation distribution but also spatial distribution of thermal dose deposition.

Recently, Liu et al. developed a portable PA device that integrates the optical module (CW laser diode with collimation lens), acoustic module (ring ultrasound transducer with an acoustic coupling layer), and back-end receiving circuit, as shown in Figure 5c–e [106]. The overall system size is around 14 cm × 8 cm × 3 cm, which is slightly larger than a normal cell phone. In vivo testing results demonstrated a high sensing accuracy of 0.5 °C, with a fast refreshing rate of 80 Hz.

### 3.4. Dosimetry for Radiation Therapy

Radiation therapy is a cancer treatment that uses a high-energy beam of X-ray radiation to kill cancerous cells or slow their growth by damaging their deoxyribonucleic acid (DNA) [107]. During X-ray radiography, both cancerous tumor and healthy tissue are susceptible to ionizing damage from irradiation; therefore, the goal of radiation therapy is to maximize the damage to a tumor while protecting healthy tissue to avoid unwanted side effects. In this sense, in vivo dosimetry is important to ensure that treatment is being delivered properly, and also has great potential for the development of adaptive radiotherapy. 

Many current clinical dosimetry techniques, including ion chambers (ICs), thermoluminescent dosimeters (TLDs), and optically stimulated luminescent dosimeters (OSLDs) require a time-consuming process, perturb the beam, or are difficult to implement [108]. The XTA technique, as a special type of EMA techniques, is intrinsically compatible with X-ray radiation therapy [109,110,111].

**Figure 6:**
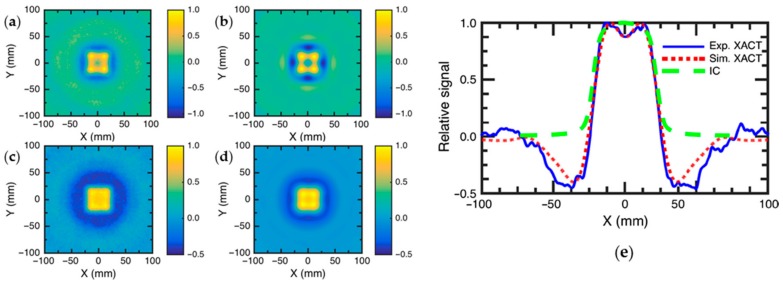
XTA imaging of radiation dose in (**a**) experiment and (**b**) simulation at a depth of 2.4 cm. XTA imaging of radiation dose in (**c**) experiment and (**d**) simulation at a depth of 10 cm; (**e**) thermodose profile estimated by simulation, experiment and ion chamber. Reprinted with permission from [109].

The characteristics of XTA dosimetry were investigated in both simulation and experimental XTA imaging for a square field at different depths [109], as shown in Figure 6. To quantitatively analyze the XTA intensity and radiation dose, amplitude profiles were extracted from experimental and simulated XTA images and compared to IC measured profiles. As shown in Figure 6e, a good agreement was observed, demonstrating the potential of XTA technique for in vivo dosimetry applications. However, this technique is still in the proof-of-concept stage and needs to be further developed towards in vivo and clinical validation.

### 3.5. Hemoglobin Oxygen Saturation (SO_2_) Sensing

As one of the most important applications of the PA techniques, PA spectroscopy detects the spectrally dependent absorption characteristics of different chromophores. In blood vessels, the absorption spectrum at visible and NIR wavelength is strongly dependent upon the concentration of oxy-hemoglobin (HbO_2_) and deoxy-hemoglobin (HbR). As shown in Figure 1, the two forms of hemoglobin have different peak absorptions in the 500–600 nm and 700–900 nm wavelength range [112]. Therefore, by acquiring PA signals at multiple wavelengths and undertaking a spectroscopic analysis, the concentration of HbO_2_, HbR, and thus calculated SO_2_ can be quantified in a manner analogous to conventional near-infrared spectroscopy (NIRs) [113].

**Figure 7:**
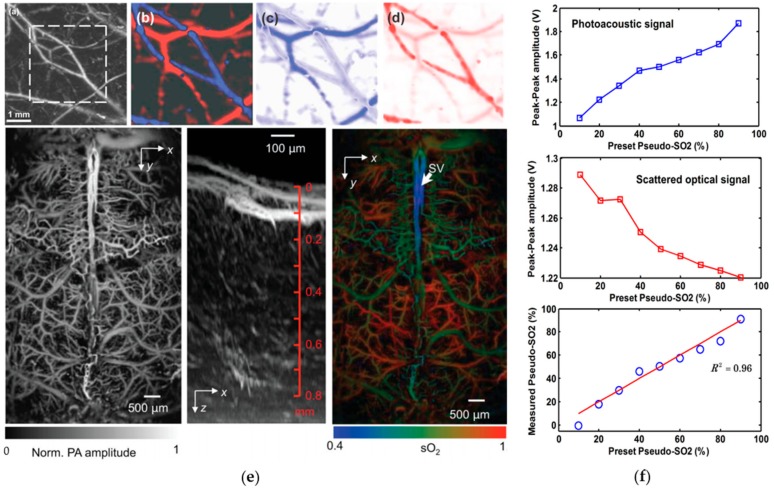
(**a**) PA microscopic imaging, (**b**) static SO_2_ image, (**c**) differential SO_2_ image (from hypoxia to normoxia), and (**d**) differential SO_2_ image (from normoxia to hypoxia) of subcutaneous blood vessels in a rat; (**e**) SO_2_ images of the mouse brain obtained by PA spectroscopic technique; (**f**) PA signal, scattered optical signal, and the measured SO_2_ level along with pseudo-SO_2_ change. Reprinted with permission from [114,115,116].

The SO_2_ level is an essential physiological parameter that is related to a broad range of pathophysiological processes such as angiogenesis, hypoxia, inflammatory, and cancerous growth. Zhang et al. investigated the principle of mapping SO_2_ distribution by four-wavelength (570, 580, 590, and 600 nm) PA microscopy in single vessels [114]. With single-wavelength PA imaging, the total hemoglobin concentration distribution was illustrated in Figure 7a. After images were acquired at all four different optical wavelengths on the same region of interest (ROI), the relative HbO_2_ and HbR were first calculated, and then the absolute SO_2_ level was calculated within the blood vessels only, based on the vessel segmentation obtained from Figure 7a. Based on the distinct SO_2_ level from normal venous and arterial blood, arteries and veins can be clearly separated, corresponding to the red and blue portions in Figure 7b, respectively. With physiological stage changing from normoxia to hyperoxia, a decreased SO_2_ level was observed (Figure 7c); from hypoxia to normoxia, the SO_2_ level increases significantly (Figure 7d). Such results were compared with those measured by optical spectrophotometer in the 700–1000 nm range, which shows a good agreement with a low difference of 4%. In [115], the SO_2_ distribution in the mouse brain is also studied using the pulse-width-based method. As shown in Figure 7e, in consonance with the oxygenation microenvironment, the averaged SO_2_ level in skull vessels was lower than that in cortical vessels. Recently, our group also proposed the single-wavelength SO_2_ detection by combining PA and optical scattering measurement [116]. Demonstrated by an in vitro experiment on porcine blood, PA and scattered light intensity are both linearly related to the HbO_2_ and HbR concentration, making it possible for SO_2_ to be measured with only one laser wavelength after calibration, as shown in Figure 7f. More reports of SO_2_ sensing based on the PA method can be found in [117] and the references therein.

### 3.6. Fingerprint Sensing

Fingerprints with unique patterns have been used as important physical evidence in healthcare biometrics to identify individuals over a long period. There has always been high demand for a simple, compact, high-resolution, fast-response, cost-effective, and nondestructive method for fingerprint detection. Inspired by PA structural imaging, Choi et al. proposed PA fingerprint detection, which combines PA imaging with acoustic impedance imaging [43]. In this method, as the imaging was conducted with the irradiation of the entire finger, high-voltage pulsed electronics in conventional ultrasonic fingerprint detection are not required.

**Figure 8:**
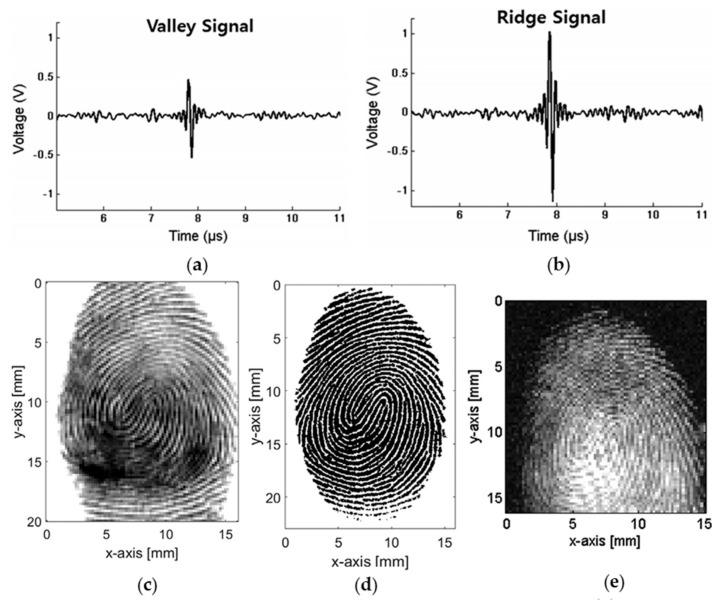
PA generation from (**a**) valley and (**b**) ridge portions of the finger; fingerprint images obtained by (**c**) ultrasonic pulse-echo impedance mismatch method, (**d**) ink-press method, and (**e**) PA method. Reprinted with permission from [43].

Due to different acoustic coupling conditions, the ridge and valley portions, i.e., the skin–acrylic and skin–air–acrylic media, could be distinguished from each other by the amplitudes of the received PA signals, as shown in Figure 8a,b. In Figure 8c–e, the fingerprint imaging results using the ultrasonic pulse-echo impedance mismatch method, ink-press method, and PA method are presented. Although the quality of the fingerprint image acquired by the PA method is slightly worse than that obtained by the ultrasound method, the PA image was sufficiently detailed to allow fingerprint pattern to be distinguished. The compact size of the PA system and potential colorimetric property of the PA mechanism may open up a new era in fingerprint detection.

### 3.7. Glucose Sensing

**Figure 9:**
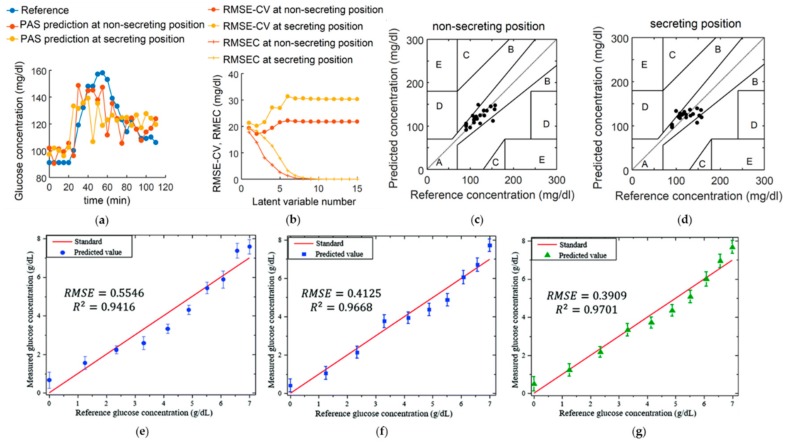
(**a**) PA measured skin glucose on the two different spots of the index finger with or without secretion from an eccrine sweat gland compared with the reference glucose level; (**b**) error analysis with varying latent variable number; (**c**,**d**) correlation between measured blood glucose and PA predicted glucose from the spectra without (**c**) or with (**d**) the secretion from a sweat gland; (**e**–**g**) correlation between predicted glucose concentration and reference concentration using (**e**) PA amplitude, (**f**) time delay, and (**g**) data fusion. Reprinted with permission from [119,120].

Blood glucose is one of the most important physiological indicators, related to a broad range of metabolic diseases (e.g., diabetes mellitus). PA-based glucose sensing has been proposed since the late 19th century [118]. However, the repeatability of such a method is highly susceptible to the skin condition, which is dependent on hand washing and drying due to the high light absorption with respect to the skin secretion products [45]. Recently, to overcome the above limitations, PA spectroscopy using mid-infrared wavelength light has been proposed [119]. From the microscopic spatial information of skin, the skin region where the infrared spectrum is insensitive to skin condition can be selected, which enables reliable prediction of the blood glucose level from the PA spectroscopic signals. Figure 9 depicts the performance of the PA glucose level sensing on two different spots with and without secretion from an eccrine sweat gland. To determine the sensing accuracy of glucose detection, Clarke’s grid was employed (Figure 9c–d). The results demonstrated that the infrared measurement on the non-secreting position produced a better prediction than that on the secreting position, with less effect from skin condition.

Later, our research group also proposed PA glucose sensing by data fusion, in which both dependencies of PA amplitude and time delay on glucose level are studied [120]. Through data fusion on the amplitude and time delay information, the accuracy of glucose sensing increased up to 33% in different glucose concentrations. To further develop PA glucose sensing for clinical practice, the sensitivity and specificity of glucose detection must be improved. One potential way is designing high-sensitivity glucose-conjugated nanoparticles as a biomarker to track the relative glucose density [121].

### 3.8. PH Sensing

Optical absorption is not only determined by the structural or molecular formation itself, but also by the environmental chemical conditions, e.g., pH level. For quantitative measurement of pH level in a biological environment, pH-sensitive nanoprobes involving fluorescence dye (SNARF-5F), sonophoric nanoprobes [46,122], albumin-based nanoprobes [123], or semiconducting oligomer nanoparticles [124] are normally required. 

**Figure 10:**
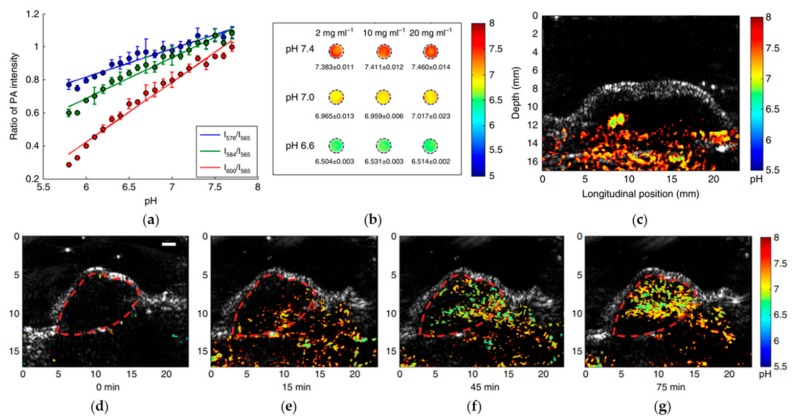
(**a**) Measured ratio of PA intensity ratios between the three wavelengths and the isosbestic point from pH 5.8–7.7; (**b**) auantitative pH images of phantoms containing different concentrations (6.6, 7.0 and 7.4); (**c**) PA pH image of a normal tissue; (**d**–**g**) auantitative PA pH images at different time points after nanoprobe injection. Reprinted with permission from [125].

In recently published quad-wavelength ratiometric PA imaging of pH [125], the PA amplitude ratios between the three wavelengths (576, 584, and 600 nm) and the isosbestic point (565 nm) were investigated. With the calibrated relationship between pH level and PA amplitude ratio (Figure 10a), PA images of the blood phantoms buffered at different pH levels are obtained and illustrated in Figure 10b. An example of PA imaging of pH level in different organs is also depicted in Figure 10c–g, in which the normal tissue shows a relative higher pH level than in cancerous tumor. Figure 10c–g shows the spatially distributed pH levels in the tumor section at different time points after injection. With the accumulation of nanoparticles in the tumor, the nanoparticle concentration and the PA amplitude reached a peak about 75 min after the injection.

Although the only optic-based PA technique is reported to measure pH level, the nanoprobe-based functional imaging method can also be extended to other EMA techniques (e.g., TA and MMTA combined with magnetic nanoparticles) for sensing other chemical-related physiological parameters (e.g., calcium [126] and potassium [127]). From another perspective of multifunctionality, by further combining EMA techniques with other therapeutic agents, the nanoprobe becomes the therapeutic nanoparticle for broader biomedical applications.

## 4. Prospects and Conclusions

Owing to their hybrid nature, EMA techniques in biomedicine offer multiple advantages over other single wave-based sensing techniques. The most favorable is the combination of excellent EM absorption contrast and acoustic-diffraction-limited spatial resolution, associated with low scattering of ultrasonic waves. EMA techniques are particularly suitable for biological tissues with inhomogeneous EM absorption but relatively homogeneous acoustical properties. Electromagnetically, inhomogeneity of EM absorption (existence of EM absorbers) provides the sensing information or imaging contrast, while the effect of physiological parameters on EM absorptions provides the functional capabilities in the EMA imaging. In this sense, the spectroscopic difference between HbO_2_ and HbR enables SO_2_ sensing, the pH-sensitive spectroscopic absorption of dyes enables pH level sensing, temperature dependence of Grüneisen parameter gives rise to the PA/TA thermometry, and so on. Acoustically, since EMA signals are generated internally by EM absorption and are propagated one way to the acoustic receiver, small speed variations within biological soft tissue do not affect the sound propagation in a finite-length path very much. Therefore, although acoustic homogeneity is preferred, EMA techniques have better tolerance to sound speed variation than conventional ultrasound techniques that rely on round-trip ultrasonic propagation.

As well as providing structural and functional information, there is the prospect of using EMA techniques for early cancer detection. The superior capabilities of EMA techniques for deep tissue imaging and the broad applications in biomedical sensing can greatly contribute to cancer detection and staging—a detailed review of cancer detection can be found in [128]. Briefly, there are two principal approaches. Firstly, cancer/tumor generation is always accompanied by physiological and metabolic changes (e.g., water content, oxygen saturation, vascular blood volume, etc.), which can be monitored from some endogenous contrasts by EMA techniques. For example, during the growth of a tumor, large amounts of oxygen and fresh nutrients need to be continuously supplied, leading to vascularization and angiogenesis around the cancerous tissue. Therefore, the malignancy of tumor can be determined based on the EMA imaging of microvasculature structure and the SO_2_ distribution [129]. Melanin, another important indicator for tumor detection (especially melanoma), shows strong optic absorption in a wide spectral range. By employing dual-wavelength PA imaging, melanomas can be spectroscopically differenced from normal tissue based on the different absorption spectrum between hemoglobin and melanin [42]. In some special situations such as breast cancer, at the margin of the tumor, microwave absorption can increase significantly due to highly increased ionic or water content, whereas the main component of the breast is fat, which shows little microwave absorption response. Therefore, the detection of breast cancer by TA is feasible and also widely studied recently [41,71,130,131]. Secondly, at the cellular level, the use of exogenous contrast in EMA has shown great promise in its ability to detect tumor vasculature [132], circulating tumor cells (CTC) [133,134,135], and micro-metastasis in sentinel lymph nodes (SNL) [136]. In PA cytometry, targeted nanoparticles are employed to label the prostate cancer cells in human blood; therefore, CTC can be easily detected with greatly enhanced contrast [133]. In a more recent study, labeled by magnetic nanoparticles, CTC can be magnetically trapped by a static magnetic field and then PA imaging can track their accumulation [135]. Using magnetic nanoparticles, such magnetic modulated PA imaging is intrinsically compatible with the MMTA method. We expect to see a combination of different EMA techniques for superior imaging and sensing performance (e.g., cellular dynamic tracking) demonstrated in the near future.

Besides EM absorption-based EMA techniques, many other EM force-related EMA methods including magnetoacoustic imaging with magnetic induction (MAT-MI) [137,138,139], magnetoacoustic imaging of superparamagnetic iron oxide nanoparticles (MAT-SPIONs), EM-induced shear wave imaging [140], magnetomotive ultrasound (MMUS) [141], and magnetomotive photoacoustic (MMPA) [142] have been developed based on their respect EM properties. In MAT-MI, a time-varying magnetic stimulation is applied to induce an eddy current in biological tissue. In the presence of a static magnetic field, the generated Lorentz force causes transient mechanical vibration and produces detectable ultrasound signals [137]. When the imaging objects are replaced by extraneous magnetic nanoparticles, the generation of a magnetoacoustic wave in MAT-SPIONs relies on the magnetic translation force [138]. In both cases, the magnetic-field-induced force serves as the acoustic source, though MAT-MI reconstructs the electrical conductivity distribution and MAT-SPIONs map the extraneous nanoparticles distribution. In MMUS/MMPA, a magnetic field is applied to induce magnetic motion within magnetically labeled tissue and ultrasound/PA is used to detect the induced tissue motion. As a hybrid phenomenon of US/PA acoustic generation and mechanical vibration, MMUS/MMPA intrinsically includes optical/acoustic contrast and mechanical contrast. Therefore, MMUS and MMPA can be used to estimate magnetic nanoparticles distribution and nanoparticle displacement in response to the magnetic field. Such dual-contrast EMA sensing techniques may further be developed for mechanical property characterization (e.g., elasticity and viscosity) and other medical applications.

In this review, we mainly focused on EM absorption-based EMA techniques. Based on the different EM absorption and heating mechanism, we categorized the most current EMA techniques into PA, TA, XA, and MMTA. In terms of biomedical applications, EMA techniques have been established as a useful tool for structural imaging (brain imaging, whole-body imaging, and molecular imaging), blood flowmetry, thermometry, dosimetry for radiation therapy, hemoglobin oxygen saturation (SO_2_) sensing, fingerprint imaging and sensing, glucose sensing, pH sensing, etc. By combining multifunctional nanoparticles, many more physiologically characteristic parameters (thermal, chemical, and mechanical) can be monitored by using EMA detection.

Overall, EMA is still in its infancy and has not yet been applied clinically, although many initial studies have demonstrated the potential for applications in the biomedical field. Several research groups are also working toward making EMA low-cost [143], stable [144], compact [145], and easy to use [146], therefore driving its translation into clinical applications. Improvement in terms of accuracy, sensitivity, penetration depth, reconstruction algorithm, and sensing speed will further improve the imaging and sensing performance of EMA. Commercialized EMA systems are expected to be available in the near future, and wide medical applications are foreseen.
